# Acute kidney injury with hypernatremia and major adverse kidney events

**DOI:** 10.1093/ckj/sfae419

**Published:** 2024-12-18

**Authors:** Jose J Zaragoza, Juan A Gómez-Fregoso, Eduardo M Hernández-Barajas, Zarahi Andrade-Jorge, Juarez Correa- de Leon, Rolando Claure-Del Granado, Jorge L Padilla-Armas, R Lizzete Ornelas-Ruvalcaba, J Said Cabrera-Aguilar, Gael Chávez-Alonso, Estefania Villalvazo-Maciel, Carlos E Orozco-Chan, Carlos B Cárdenas-Mercado, Gonzalo Rodríguez-García, Guillermo Navarro-Blackaller, Ramón Medina-González, Alejandro Martínez Gallardo-González, Luz Alcantar-Vallin, Gabriela J Abundis-Mora, Guillermo García-García, Jonathan S Chávez-Iñiguez

**Affiliations:** Intensive Care Unit, Hospital H+ Queretaro, Mexico; Nephrology Service, Hospital Civil de Guadalajara Fray Antonio Alcalde, Guadalajara, Jalisco, Mexico; University of Guadalajara Health Sciences Center, Guadalajara, Jalisco, Mexico; Nephrology Service, Hospital Civil de Guadalajara Fray Antonio Alcalde, Guadalajara, Jalisco, Mexico; University of Guadalajara Health Sciences Center, Guadalajara, Jalisco, Mexico; Nephrology Service, Hospital Civil de Guadalajara Fray Antonio Alcalde, Guadalajara, Jalisco, Mexico; University of Guadalajara Health Sciences Center, Guadalajara, Jalisco, Mexico; Nephrology Service, Hospital Civil de Guadalajara Fray Antonio Alcalde, Guadalajara, Jalisco, Mexico; University of Guadalajara Health Sciences Center, Guadalajara, Jalisco, Mexico; Division of Nephrology, Hospital Obrero No. 2 – Caja Nacional de Salud, Cochabamba, Bolivia; IIBISMED, Facultad de Medicina, Universidad Mayor de San Simón, Cochabamba, Bolivia; Nephrology Service, Hospital Civil de Guadalajara Fray Antonio Alcalde, Guadalajara, Jalisco, Mexico; University of Guadalajara Health Sciences Center, Guadalajara, Jalisco, Mexico; Nephrology Service, Hospital Civil de Guadalajara Fray Antonio Alcalde, Guadalajara, Jalisco, Mexico; University of Guadalajara Health Sciences Center, Guadalajara, Jalisco, Mexico; Nephrology Service, Hospital Civil de Guadalajara Fray Antonio Alcalde, Guadalajara, Jalisco, Mexico; University of Guadalajara Health Sciences Center, Guadalajara, Jalisco, Mexico; University of Guadalajara Health Sciences Center, Guadalajara, Jalisco, Mexico; University of Guadalajara Health Sciences Center, Guadalajara, Jalisco, Mexico; University of Guadalajara Health Sciences Center, Guadalajara, Jalisco, Mexico; University of Guadalajara Health Sciences Center, Guadalajara, Jalisco, Mexico; Nephrology Service, Hospital Civil de Guadalajara Fray Antonio Alcalde, Guadalajara, Jalisco, Mexico; Nephrology Service, Hospital Civil de Guadalajara Fray Antonio Alcalde, Guadalajara, Jalisco, Mexico; University of Guadalajara Health Sciences Center, Guadalajara, Jalisco, Mexico; Nephrology Service, Hospital Civil de Guadalajara Fray Antonio Alcalde, Guadalajara, Jalisco, Mexico; Nephrology Service, Hospital Civil de Guadalajara Fray Antonio Alcalde, Guadalajara, Jalisco, Mexico; University of Guadalajara Health Sciences Center, Guadalajara, Jalisco, Mexico; Nephrology Service, Hospital Civil de Guadalajara Fray Antonio Alcalde, Guadalajara, Jalisco, Mexico; University of Guadalajara Health Sciences Center, Guadalajara, Jalisco, Mexico; Nephrology Service, Hospital Civil de Guadalajara Fray Antonio Alcalde, Guadalajara, Jalisco, Mexico; University of Guadalajara Health Sciences Center, Guadalajara, Jalisco, Mexico; Nephrology Service, Hospital Civil de Guadalajara Fray Antonio Alcalde, Guadalajara, Jalisco, Mexico; University of Guadalajara Health Sciences Center, Guadalajara, Jalisco, Mexico

**Keywords:** acute kidney injury, hypernatremia, major adverse kidney events

## Abstract

**Introduction:**

Consequences of hypernatremia in akute kidney injury (AKI-hyperNa) is poorly understood. We analyzed the risk of major adverse kidney events (MAKE) in comparison with AKI and normal serum sodium (AKI-normalNa). Such data could help in understanding this complex interaction.

**Methods:**

In this retrospective cohort we compared the AKI-hyperNa with the AKI-normalNa regarding the risk of MAKE, which include death, new dialysis requirement, and worsening kidney function (≥25% decrease in estimated glomerular filtration rate), at 10 (MAKE10) and at 30–90 days (MAKE30–90) using multivariate logistic regression and area under the curve (AUC) analysis. The association between serum sodium levels (per 1 mEq/l increase) and the number of days with hypernatremia was also compared.

**Results:**

A total of 357 patients were included (78 with AKI-hyperNa and 279 with AKI-normalNa). Compared to the AKI-normalNa, AKI-hyperNa were predominantly male (73% versus 59%), experienced hypernatremia for a longer duration (3 days versus 0 days), and took 5 days to normalize serum sodium levels. After multivariate regression analysis, the AKI-hyperNa group had higher risk of MAKE10 [odds ratio (OR) 5.7, confidence interval (CI) 2.5 to 12.89, *p* < 0.001] with an AUC of 0.79. Also its components such as mortality and decreased estimated glomerular filtration rate (OR 2.13, CI 1.11 to 4.07, *p* = 0.021 and OR 20.14, CI 7.69 to 10.03, *p* = 0.001, respectively). A similar trend was found for MAKE30–90 (OR 4.17, CI 1.73 to 10.03, *p* ≤ 0.001). A gradual increase in serum sodium was associated with a higher risk of MAKE (OR 1.07, CI 1.04 to 1.11, *p* ≤ 0.001), as was the number of days spend with hypernatremia (OR 1.51, CI 1.22 to 1.87, *p* = 0.001).

**Conclusions:**

In this cohort, AKI-hyperNa compared with AKI-normalNa had a fivefold risk of short- and long-term MAKE. This event was more frequently observed as serum sodium increased and it was closely related to the number of days that hypernatremia persisted.

KEY LEARNING POINTS

- **What was known**: Acute kidney injury is a risk factor to develop hypernatremia, the clinical trajectory of this in patients is not well understood.
- **This study adds**: AKI with hypernatremia, when compared with AKI patients without hypernatremia, had a higher risk of presenting major adverse kidney events, such as the need of dialysis, death or kidney function deterioration. In addition, we found that the more the serum sodium value increases and the more days that hypernatremia persists, the higher the risk of these complications.
- **Potential impact**: Our results would help to better understand and identify these patients, contributing to promoting the prevention of hypernatremia and normalizing serum sodium values in acute kidney injury.

## INTRODUCTION

Hypernatremia, defined as a plasma sodium concentration of >145 mEq/l, occurs due to a net gain of water-free electrolytes or a net loss of electrolyte-free water. The kidneys must perform aquaresis (approximately 400–500 ml/day) to excrete urinary osmoles, while central osmoregulation adjusts the remaining urinary water loss to maintain overall water balance and plasma osmolality, provided there is hemodynamic stability [1].

Kidney function is unsurprisingly the most critical determinant in the development of hypernatremia [2] commonly observed in patients with acute kidney injury (AKI) due to dysfunctional solute and water homeostasis [3]. However, the relationship between AKI and hypernatremia may be bidirectional and synergistic, with hypernatremia increasing the risk of AKI and AKI increasing the risk of hypernatremia [4]. In this context, the kidneys generally have a reduced capacity to regulate water and sodium homeostasis, and the coexistence of both conditions significantly heightens the risk of poor clinical outcomes [5, 6].

Although hypernatremia frequently occurs in patients with AKI, it remains poorly understood, and its prognosis is believed to be worse than that of patients with normal serum sodium levels. There are no specific treatments available to enhance kidney function recovery after an AKI episode or to reduce mortality risk [7], making it essential to understand and manage complications like hypernatremia. Further investigation is needed to explore the characteristics and clinical course of patients with AKI and hypernatremia (AKI-hyperNa). To address this problem, we analyzed a cohort of patients to evaluate the clinical characteristics of individuals with AKI-hyperNa and assess the risk of major adverse kidney events (MAKE) during follow-up, compared with patients with AKI and normal serum sodium levels (AKI-normalNa). These findings would help to clarify this complex interaction and improve care for this population.

## MATERIALS AND METHODS

### Study design and patient population

In this retrospective cohort study, we enrolled patients admitted to the Civil Hospital of Guadalajara Fray Antonio Alcalde, Mexico. All patients included in the study were under the care of either a primary medical or surgical team. The patients were in the intensive care units and in the general hospital wards. We focused on patients diagnosed with AKI who received a nephrology consultation. These patients were assessed at the time of their initial AKI diagnosis and subsequently followed up in the clinic. A 10-day hospital follow-up period was chosen, as most patients with AKI typically commence kidney replacement therapy (KRT) within this time frame [8].

The cohort was divided into two groups for the analysis: the group of interest was those with AKI-hyperNa (AKI and serum sodium >145 mmol/l) and the comparison group was AKI-normalNa (AKI and serum sodium <145 mmol/l including those with hyponatremia). The serum sodium index value was considered when the diagnosis of AKI was made. Patients who had <3 serum sodium measurements were excluded.

The diagnosis of AKI was based on the serum creatinine (sCr) criteria as defined by the Kidney Disease: Improving Global Outcomes (KDIGO) guidelines [9]. We did not consider urine output because it was not accurately collected in some patients. The baseline sCr level was defined as the most recent value obtained within the year prior to admission. For patients without available baseline sCr values, the level was estimated using the Modification of Diet in Renal Disease equation, assuming an estimated glomerular filtration rate (eGFR) of 75 ml/min/1.73 m² [9]. We included only patients who had recorded admission serum sodium levels and at least three sCr measurements during the follow-up period. Exclusion criteria included patients with chronic kidney disease (CKD) stage 5, defined as an eGFR of <15 ml/min/1.73 m² using the CKD-Epidemiology Collaboration equation [10], patients on chronic dialysis, those hospitalized for less than 48 hours, all transplant recipients, pregnant patients, and cases with incomplete data that precluded analysis.

### Data collection

Clinical characteristics, demographic information, and laboratory data were collected through automated retrieval from the institutional electronic medical record system. The demographic and clinical data collected included age, diabetes status, hypertension status, hypothyroidism status, CKD grade, smoking status, cerebrovascular disease status, and incidence of ischemic heart disease. Serum sodium levels were measured using direct ion-selective potentiometry with an AU5800 chemical analyzer (Beckman Coulter, Inc.®). In-hospital serum sodium levels were assessed based on the values obtained during the hospital stay, although some values were missing due to sporadic blood sample collection. Serum sodium levels were not adjusted for glucose levels. Contributing factors to AKI, such as sepsis (defined by the Sepsis-3 criteria) [11], clinical hypovolemia [12], cardiorenal syndrome [13], nephrotoxic drugs, and shock, were prespecified. Additionally, biochemical data including hemoglobin, platelets, leukocytes, glucose, urea, sCr, sodium, potassium, chloride, phosphate, calcium, arterial pH, PCO_2_, PO_2_, bicarbonate, and lactate levels were also recorded. Fluid administration and fluid volume adjustments were determined using objective metrics derived from point-of-care ultrasound evaluations performed by the nephrology team [14, 15]. Fluid overload was defined when >5 B-lines in lungs or a VExUS score >1 was documented. In most cases, nephrotoxic drugs and antibiotics were dose-adjusted and administered in collaboration with the nephrology team. Indications for KRT included fluid overload resistant to diuretics, severe hyperkalemia and severe metabolic acidosis refractory to medical treatment, and uremic manifestations such as encephalopathy, pericarditis, and seizures [16]. We assessed MAKE as defined by the National Institute of Diabetes and Digestive and Kidney Disease workgroup for the study of AKI [17]. MAKE includes three clinically significant outcomes that reflect both short- and long-term patient-centered outcomes: death, new requirement for dialysis, and worsening of kidney function, defined as a ≥25% decrease in eGFR from baseline [18]. To provide a comprehensive follow-up, we assessed the frequency and risk of MAKE at 10 days (MAKE10) and at 30–90 days (MAKE30–90).

The study was approved by the Hospital Civil de Guadalajara Fray Antonio Alcalde Institutional Review Board (HCG/CEI-0550/15) and was conducted according to the Declaration of Helsinki [19]. In adherence to the ethical standards outlined in the Declaration of Helsinki, informed consent was not required for this study. The study protocol was designed to align with the Strengthening the Reporting of Observational Studies in Epidemiology (STROBE) guidelines [20] and the REporting of studies Conducted using Observational Routinely collected health Data (RECORD) statement [21].

### Study outcomes

The primary outcome was to compare patients with AKI-hyperNa with AKI-normalNa in the risk of MAKE10. As secondary outcomes, we considered each of the MAKE components at 10 days, MAKE30–90, a granular association between serum sodium per 1 mEq/l increase and days spent with hyperNa with the risk of MAKE.

### Statistical analysis

The distribution of quantitative variables was examined visually using histograms, and the Kolmogorov–Smirnov and Shapiro–Wilk tests were used to confirm their non-normal distribution. The evolution of serum sodium levels is represented by box plots from day 1 to day 10 of the hospital stay, and differences between the groups were analyzed using the Wilcoxon test. Continuous variables are summarized as medians and interquartile ranges, while categorical variables are expressed as counts, proportions, or percentages. Groups were created according to the sodium status (hypernatremia and non-hypernatremia). Differences in categorical variables between the groups were analyzed using the χ^2^ test or Fisher's exact test, as appropriate. Continuous variables were compared using the Wilcoxon rank test. Multivariate logistic regression analysis was performed to determine the risk of MAKE. We included every variable when *p* was <0.05 in the bivariate analysis and added hypernatremia as a dichotomous variable. The coefficient plot and receiver operating characteristic curve for the entire model are presented. Logistic regression multivariate analysis was replicated for secondary outcomes (death, KRT, >25% decrease in eGFR, and MAKE at days 30–90). The predictive margins of probability plot from the logistic regression model were represented for every 2-mmol increase in serum sodium and for the number of days that the patients remained in hypernatremia. Missing data were handled by imputation with the median or mean; each missing data was replaced with the value predicted by a regression model. Statistical significance was defined as *p* < 0.05. Data were analyzed using Stata version 16.1 (StataCorp, College Station, TX, USA).

## RESULTS

**Figure 1:**
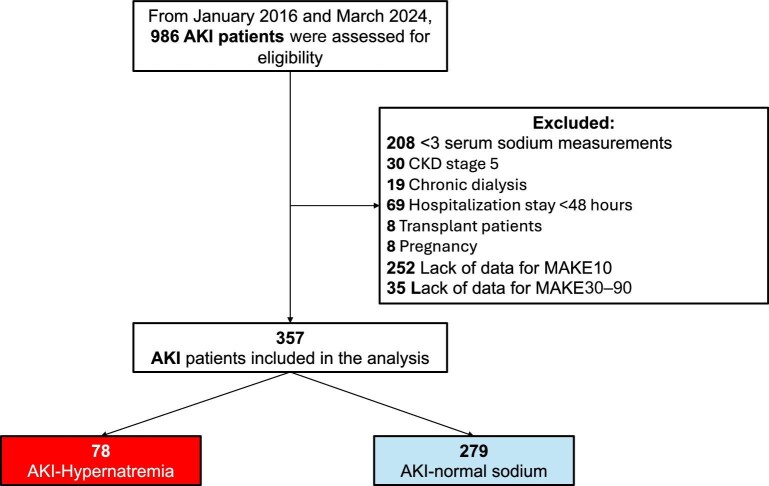
Flowchart of study population.

From January 2016 to March 2024, a total of 986 patients with AKI were assessed for eligibility. Of these, 629 were excluded for not meeting the inclusion criteria, leaving 357 patients for the final analysis. Among them, 78 patients had AKI-hyperNa, and 279 had AKI-normalNa at the time of the initial nephrology evaluation. A flowchart of the study cohort is presented in Fig. 1.

### Demographic baseline characteristics

Table 1 shows the baseline demographics and clinical characteristics of patients with AKI according to the presence of hypernatremia at inclusion. When compared to the AKI-normalNa group, patients with AKI-hyperNa were predominantly male (73% versus 59%), had higher systolic blood pressure (119 mmHg versus 114 mmHg), higher body temperature (36.7°C versus 36.5°C), and a higher heart rate (90 bpm versus 85 bpm). In managing AKI, the AKI-hyperNa group required more frequent fluid and antibiotic dose modifications, nutritional adjustments, and fluid changes than the AKI-normalNa group (*p* ≤ 0.05 for all comparisons).

**Table 1:** Baseline clinical characteristics of patients according to hypernatremia at nephrology evaluation.

Variable	AKI-normalNa (Na^+^ < 145 mmol/l) n = 279	AKI-hyperNa (Na^+^ > 145 mmol/l) n = 78	Total	P value
**Demographic characteristics**				
Age (years)	54 (40–67)	55 (41–68)	55 (40–67)	0.909
Male sex (%)	166 (59.5)	57 (73.08)	223 (62.46)	0.034^a^
Weight (kg)	70 (60–80)	75 (67.5–81.25)	70 (62–80)	0.005^a^
Height (m)	1.65 (1.59–1.71)	1.69 (1.64–1.74)	1.65 (1.6–1.72)	0.003^a^
Systolic pressure (mmHg)	114 (101–129)	119 (108–137)	115 (102–130)	0.040^a^
Diastolic pressure (mmHg)	68 (59–78)	71.5 (61–80)	69 (60–78)	0.098
Temperature (C^o^)	36.5 (36–36.9)	36.7 (36.3–37.4)	36.5 (36–37)	0.003^a^
Heart rate	85 (76–96)	90.5 (80–101)	86 (77–98)	0.019^a^
Respiratory rate	19 (17–20)	19 (17–22)	19 (17–20)	0.173
Diabetes (%)	82 (23.39)	17 (21.79)	99 (27.73)	0.201
Hypertension (%)	86 (30.82)	28 (35.9)	114 (31.93)	0.412
Tobacco use (%)	62 (22.22)	12 (15.38)	74 (20.73)	0.209
Hypothyroidism (5)	4 (1.43)	0	4 (1.12)	0.580
Congestive heart failure (%)	30 (10.75)	5 (6.41)	35 (9.80)	0.291
CKD (%)	26 (9.32)	5 (6.41)	31 (8.68)	0.502
Ischemic stroke (%)	7 (2.51)	5 (6.41)	12 (3.36)	0.145
Coronary artery disease (%)	15 (5.38)	1 (1.28)	16 (4.48)	0.211
Cirrhosis (%)	13 (4.66)	1 (1.28)	14 (3.92)	0.319
NSAIDs (%)	79 (28.32)	18 (23.08)	97 (27.17)	0.391
Antibiotic use (%)	180 (64.52)	60 (76.92)	240 (67.23)	0.041
Antihypertensive use (%)	68 (24.37)	20 (25.64)	88 (24.65)	0.882
Diuretic use (%)	95 (34.05)	23 (29.49)	118 (33.05)	0.498
Vasopressor use (%)	89 (31.9)	27 (34.62)	116 (32.49)	0.196
Statin use (%)	43 (15.4)	7 (8.97)	50 (14.01)	0.148
Aspirin (%)	27 (9.68)	3 (3.85)	30 (8.40)	0.111
ISGLT2	26 (9.32)	1 (1.89)	27 (8.13)	0.096
Baseline creatinine (mg/dL)	0.9 (0.7–1.09)	0.92 (0.74–1.06)	0.9 (0.7–1.08)	0.544
Days with hypernatremia	0 (0–0)	3 (2–6)	0 (0–0)	0.000^a^
eGFR (ml/min/1.73m^2^)	17.88 (9.92–27.3)	18.7 (10.7–32.7)	17.9 (10.04–27.7)	0.476
AKI Stage 1	18 (6.45)	2 (2.56)	20 (5.60)	0.267
AKI Stage 2	36 (12.90)	12 (15.28)	48 (13.45)	0.575
AKI Stage 3	224 (80.29)	61 (78.21)	285 (78.83)	0.685
Acute on chronic AKI	1 (0.36)	3 (3.85)	4 (1.12)	0.034^a^
**AKI Etiology (%)**				
Sepsis	131 (46.95)	41 (52.56)	172 (48.18)	0.442
Hypovolemia	73 (26.16)	22 (28.21)	95 (26.61)	0.772
Cardiorenal syndrome	28 (10.04)	4 (5.13)	32 (8.96)	0.261
Nephrotoxic medication	18 (6.45)	2 (2.56)	20 (5.60)	0.267
Shock state	57 (20.43)	13 (16.67)	70 (19.61)	0.521
Obstruction	41 (14.70)	6 (7.69)	47 (13.17)	0.130
Hyperbilirubinemia	5 (1.79)	0	5 (1.40)	0.590
**Treatment (%)**				
Fluid adjustment	259 (92.83)	56 (83.58)	315 (91.04)	0.029^a^
Nephrotoxic suspension	19 (6.81)	9 (13.43)	28 (8.09)	0.083
Antibiotic adjustment	29 (10.39)	14 (20.90)	43 (12.43)	0.024^a^
Nutritional adjustment	2 (0.72)	4 (5.97)	6 (1.74)	0.014^a^
Change to non-hyperchloremic fluids	4 (1.43)	7 (10.45)	11 (3.18)	0.001^a^
**AKI complication (%)**				
Hyperkalemia	33 (11.83)	8 (10.26)	41 (11.48)	0.842
Metabolic Acidosis	23 (8.24)	12 (15.38)	35 (9.80)	0.082
Fluid overload	20 (7.17)	7 (8.97)	27 (7.56)	0.629
Uremia	34 (12.19)	14 (17.95)	48 (13.45)	0.192
**Laboratory values**				
Hemoglobin (g/dl)	9.81 (8.24–11.69)	10.12 (8.61–11.5)	9.92 (8.29–11.69)	0.588
Platelets (× 10^9^/l)	213 (128.6–323.1)	179 (114.8–275.7)	207 (125.4–314.8)	0.213
Leucocytes (× 10^9^/l)	12.59 (8.69–16.82)	13.14 (8.54–20.71)	12.59 (8.62–18.1)	0.278
Glucose (mg/dl)	109 (86–147)	126 (99–171)	112 (88–156)	0.051
Urea (mg/dl)	131 (94–193)	169 (113–204)	136 (96–201)	0.013^a^
Creatinine (mg/dl)	3.42 (2.2–5.36)	3.38 (2.36–5.1)	3.41 (2.22–5.23)	0.870
Sodium (mEq/L)	134 (128–138)	150 (147–153)	135 (130–142)	0.000^a^
Potassium (mEq/L)	4.76 (4.09–5.65)	4.49 (3.72–5.06)	4.63 (4–5.56)	0.007^a^
Chloride (mEq/L)	101 (96–106)	117 (109.3–120)	103 (98–109)	0.000^a^
Calcium (mg/dl)	7.9 (7.3–8.5)	7.9 (7.3–8.6)	7.9 (7.3–8.5)	0.603
Phosphorus (mg/dl)	5.7 (4.5–7.6)	5 (3.6–6.7)	5.6 (4.2–7.4)	0.002^a^
At least one day with hyperNa	176 (63.08)	78 (100)	254 (71.15)	0.000^a^

Abbreviations: CKD: chronic kidney disease; HR: Hazard ratio; LCI: lower confidence interval; MAKE: major adverse kidney events; NSAIDs: Non steroidal analgesic drugs; SGLT2i: sodium glucose type 2 inhibitors; UCI: upper confidence interval.

a p= <0.05

The AKI-hyperNa group had higher values of urea (169 mg/dl versus 131 mg/dl) and chloride (117 mEq/l versus 101 mEq/l) and less potassium (4.49 mEq/l versus 4.76 mEq/l) than those in the AKI-normalNa group (*p* ≤ 0.05 for all). The serum sodium trajectories according to the groups are presented in Supplemental Table 1 and Supplemental Fig. 1. Compared with AKI-normalNa, the AKI-hyperNa group had hypernatremia for a longer duration (3 days versus 0 days), had more frequently at least 1 day with hypernatremia (100% versus 63%), had higher serum sodium values throughout the first 10 days (*p* ≤ 0.05 for all), and took 5 days to normalize serum sodium (150–143 mmol/l).

The frequencies of the outcomes are presented in Table 2. A total of 225 patients experienced immediate MAKE, and 166 patients died. Compared to the AKI-normalNa, the AKI-hyperNa group had a higher frequency of MAKE10 (83.3% versus 57.3%) and all its individual components, including mortality (64.1% versus 41.58%), decreased eGFR (70.5% versus 39.7%), new dialysis requirement (39.7% versus 25.8%) (*p* < 0.05 for all comparisons). A similar trend was observed for MAKE30–90 (88.4% versus 70.6%) and the mortality component at 30–90 days (78.2% versus 61.6%).

**Table 2:** Clinical outcomes according to the AKI groups.

Variable	AKI-normalNa (Na^+^ < 145 mmol/l)	AKI-hyperNa (Na^+^ > 145 mmol/l)	Total	P value
MAKE at 10 (%)	160 (57.3)	65 (83.3)	225 (63.03)	0.000^a^
Mortality (%)	116 (41.58)	50 (64.1)	166 (46.5)	0.000^a^
Decrease eGFR	111 (39.7)	55 (70.51)	166 (46.5)	0.000^a^
KRT (%)	72 (25.8)	31 (39.74)	103 (28.8)	0.016^a^
IDH	47 (16.85)	26 (33.3)	73 (20.4)	0.001^a^
PD	5 (1.79)	3 (3.85)	8 (2.24)	0.380
CRRT	20 (7.17)	2 (2.56)	22 (6.16)	0.135
MAKE at 30–90 (%)	197 (70.61)	69 (88.46)	266 (74.51)	0.001^a^
Mortality at 30–90 (%)	172 (61.6)	61 (78.21)	233 (65.2)	0.007^a^
Decrease GFR at 30–90 (%)	20 (7.17)	4 (5.13)	24 (6.72)	0.619
KRT at 30–90 (%)	83 (29.7)	32 (41)	115 (32.2)	0.060

Abbreviations: **MAKE: major adverse kidney events. HR: Hazard ratio. LCI: lower confidence interval. UCI: upper confidence interval.**

a **p= <0.05**

### Primary and secondary outcomes

**Figure 2:**
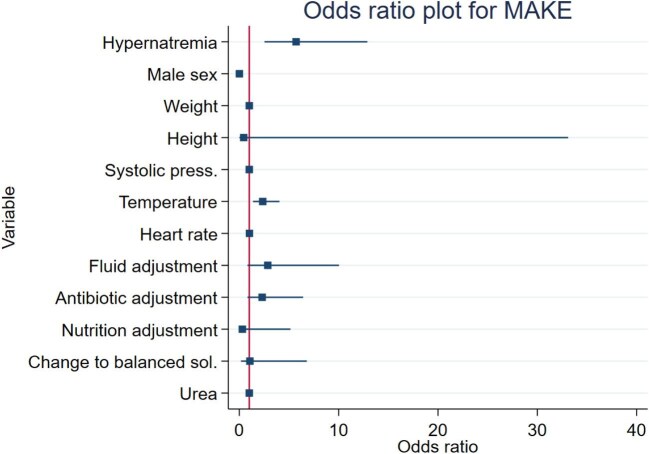
Forest plot for the OR of MAKE10.

The results of the primary objectives are presented in Table 3. After adjusted multivariate analysis, the AKI-hyperNa group demonstrated a 5.7-fold increased risk of developing MAKE10 (OR 5.7, CI 2.5 to 12.89, *p* < 0.001). This was the variable with the strongest association with this complication, as shown by the forest plot in Fig. 2. The area under the curve (AUC) for AKI-hyperNa as a predictor of MAKE10 was 0.79, indicating good predictive value (Supplemental Fig. 2). Other associated variables included body temperature and male sex.

**Table 3:** Logistic regression model for MAKE10.

MAKE	OR	LCI	UCI	P
Hypernatremia	5.722	2.540	12.892	<0.001*
Male sex	0.125	0.003	0.040	<0.001*
Weight	1.003	0.982	1.024	0.736
Height	0.441	0.005	33.09	0.711
Systolic pressure	1.003	0.987	1.019	0.699
Temperature	2.360	1.377	4.044	0.002*
Heart rate	1.016	0.998	1.035	0.072
Fluid adjustment	2.869	0.820	10.03	0.099
Antibiotic adjustment	2.303	0.826	6.423	0.111
Nutrition adjustment	0.309	0.018	5.146	0.413
Change to no hyperchloremic fluid	1.064	0.166	6.795	0.947
Urea	1.003	0.999	1.007	0.060

Abbreviations: **MAKE: major adverse kidney events. OR: Odds ratio. LCI: lower confidence interval. UCI: upper confidence interval.**

**Figure 3:**
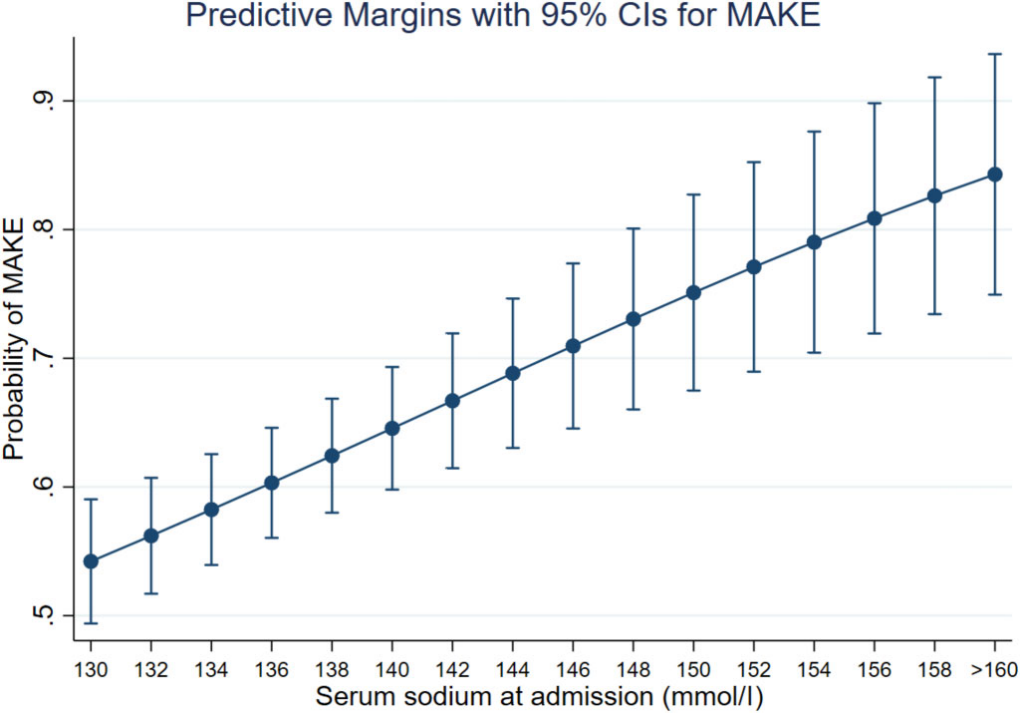
Predictive margins of MAKE by serum sodium at nephrology evaluation.

Supplemental Table 2 and Fig. 3 show that a gradual increase in serum sodium values (per 1 mEq/l) was associated with a higher risk of MAKE (OR 1.077, CI 1.040 to 1.116, *p* ≤ 0.001), MAKE was observed in >80% of the cases where the serum sodium level >156 mEq/l. We observed an association between MAKE and the number of days hypernatremia was maintained (OR 1.511, CI 1.222 to 1.870, *p* = 0.001; Supplemental Table 3). The higher the number of days with hypernatremia, the more likely the occurrence of MAKE. With >5 days of hypernatremia, 80% of the patients experienced this event (Supplemental Fig. 3). The AKI-hyperNa group had a higher risk of presenting MAKE10 components separately, such as death and decrease in eGFR (OR 2.136, CI 1.119 to 4.077, *p* = 0.021 and OR 20.14, CI 7.695 to 10.03, *p* = 0.001, respectively), but no association was found with new dialysis requirement (Table 4).

**Table 4:** Logistic regression model for each of the components of MAKE10 by the presence of hypernatremia.

Death	OR	LCI	UCI	P
Hypernatremia	2.136	1.119	4.077	0.021*
Male sex	0.840	0.440	1.604	0.598
Weight	0.997	0.983	1.012	0.750
Height	14.78	0.466	468.1	0.127
Systolic pressure	1.006	0.993	1.019	0.305
Temperature	1.834	1.191	2.823	0.006*
Heart rate	1.015	1.000	1.029	0.038*
Fluid adjustment	4.289	1.452	12.67	0.008*
Antibiotic adjustment	1.852	0.825	4.154	0.135
Nutrition adjustment	5.548	0.264	116.2	0.270
Change to no hyperchloremic fluid	0.168	0.017	1.593	0.120
Urea	1.002	0.999	1.005	0.097
KRT	OR	LCI	UCI	P

Hypernatremia	1.231	0.616	2.460	0.555
Male sex	0.892	0.393	1.748	0.623
Weight	0.995	0.978	1.012	0.597
Height	1.414	0.025	78.37	0.866
Systolic pressure	1.012	0.998	1.027	0.086
Temperature	1.542	0.984	2.418	0.059
Heart rate	1.027	1.011	1.044	0.001*
Fluid adjustment	3.991	1.213	13.13	0.023*
Antibiotic adjustment	3.470	1.532	7.857	0.003*
Nutrition adjustment	1.652	0.178	15.32	0.658
Change to no hyperchloremic fluid	0.822	0.120	5.631	0.842
Urea	1.005	1.002	1.009	0.001*
Decrease in eGFR	OR	LCI	UCI	P

Hypernatremia	20.14	7.695	52.72	<0.001*
Male sex	0.005	0.001	0.018	<0.001*
Weight	0.979	0.954	1.006	0.130
Height	0.324	0.001	101.4	0.701
Systolic pressure	0.997	0.978	1.017	0.807
Temperature	0.888	0.465	1.696	0.720
Heart rate	1.008	0.987	1.030	0.424
Fluid adjustment	0.777	0.156	3.864	0.758
Antibiotic adjustment	0.378	0.093	1.539	0.175
Nutrition adjustment	2.150	0.112	41.01	0.611
Change to no hyperchloremic fluid	0.665	0.066	6.690	0.730
Urea	1.002	0.997	1.007	0.282

Abbreviations: **MAKE: major adverse kidney events. OR: Odds ratio. LCI: lower confidence interval. UCI: upper confidence interval.**

To evaluate the impact on long-term MAKE in the AKI-hyperNa group, a logistic regression model was performed for MAKE30–90, which showed that hypernatremia was associated with a fourfold increase in risk (OR 4.174, CI 1.736 to 10.03, *p* ≤ 0.001; Table 5).

**Table 5:** Logistic regression model for MAKE30-90.

MAKE at day 30–90	OR	LCI	UCI	P
Hypernatremia	4.174	1.736	10.03	0.001*
Male sex	0.028	0.007	0.103	<0.001*
Weight	0.998	0.978	1.017	0.854
Height	0.039	0.001	2.283	0.118
Systolic pressure	0.997	0.982	1.012	0.746
Temperature	2.047	1–186	3.532	0.010*
Heart rate	1.014	0.996	1.033	0.107
Fluid adjustment	2.062	0.612	6.944	0.243
Antibiotic adjustment	1.447	0.495	4.226	0.499
Change to no hyperchloremic fluid	0.550	0.078	3.861	0.548
Urea	1.002	0.999	1.006	0.142

Abbreviations: **MAKE: major adverse kidney events. OR: Odds ratio. LCI: lower confidence interval. UCI: upper confidence interval.**

## DISCUSSION

**Figure 4:**
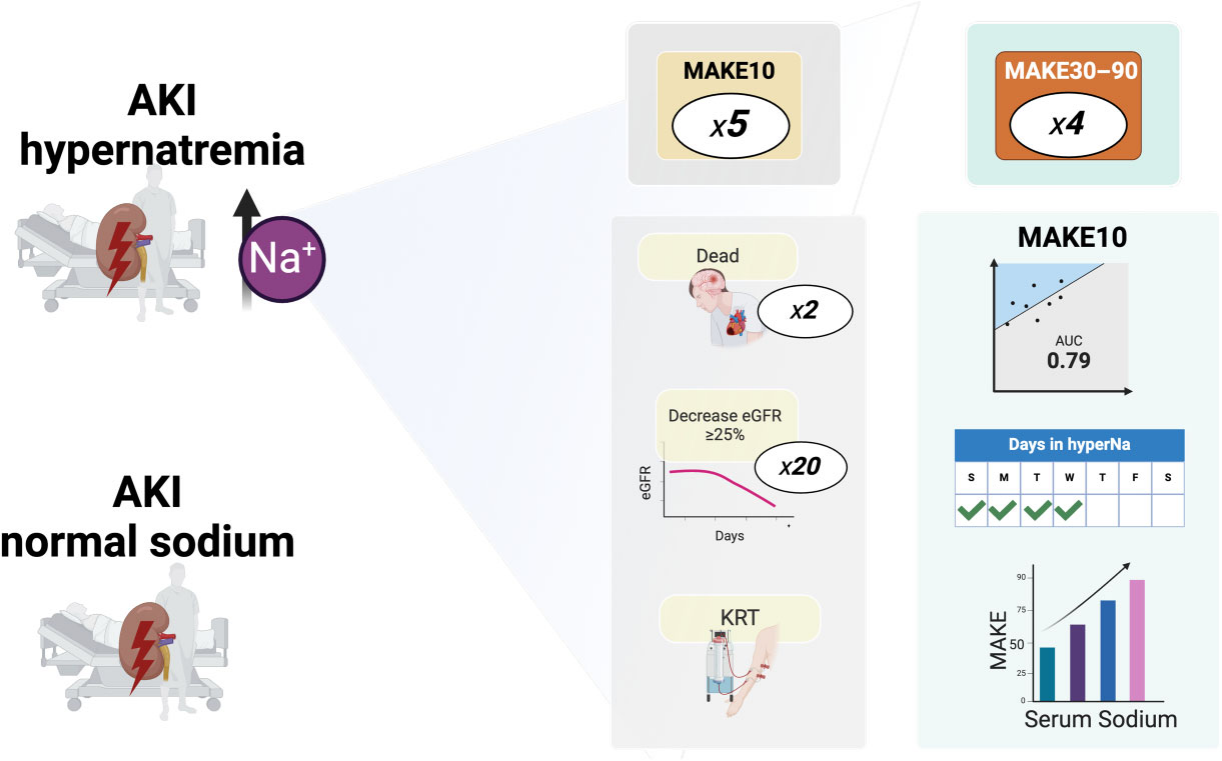
Central figure.

In this cohort of patients with AKI, those who developed hypernatremia on the first day of evaluation were found to have a fivefold increased risk of both short- and long-term MAKE. The frequency of this event also increased as serum sodium levels rose, even within the normal range, and was closely associated with the duration of hypernatremia (see Fig. 4 and the Graphical Abstract).

The prevalence of hypernatremia in our cohort of patients with AKI was 12%, which differs from the 1–4% prevalence typically seen in the general hospitalized population (not specifically those with AKI) [5]. However, in intensive care units, the prevalence of hypernatremia can reach up to 26% [22]. The mortality rate in the AKI-hyperNa group was nearly 80%, consistently higher across all evaluations compared to the AKI-normalNa group. The increased likelihood of death in patients with AKI-hyperNa observed in our cohort is consistent with previous findings linking hypernatremia with AKI. Chávez-Íñiguez *et al*. reported that patients with persistent hypernatremia during the first 10 days of hospitalization had a threefold higher risk of death, which diminished when serum sodium levels normalized [13]. Another study found that patients who died with AKI had higher serum sodium levels than survivors (145 mEq/l versus 138 mEq/l, *p* = 0.003) [6]. Additionally, a study of nearly 2 million hypernatremic patients reported an in-hospital mortality rate of 12%, compared to 2% in normonatremic patients [5]. In a cohort of over 655 000 patients with CKD, 1.9% had hypernatremia and a 33% increased risk of death compared to those with normal serum sodium levels [23].

The association between acute hypernatremia and mortality has several potential explanations. Hypernatremia can cause cellular shrinkage, leading to intracranial hemorrhage, seizures, coma, and osmotic demyelination syndrome, which may present as central pontine and extrapontine myelinolysis [23]. In the cardiovascular system, hypernatremia is associated with negative chronotropic effects and myocardial dysfunction [22, 24]. The lack of association between AKI-hyperNa and the initiation of KRT when analyzed individually is striking. We believe that this is because in the ‘real world’ most known indications for the initiation of KRT in AKI do not contemplate hyperNa.

The AKI-hyperNa group did not show more long-term kidney events, such as new dialysis requirement or a decrease in eGFR, likely because the increase in sodium was acute and did not persist long enough to promote renal fibrosis and long-term functional deterioration, as seen in chronic hypernatremia [25].

Several mechanisms may explain the complex interaction between AKI and hypernatremia: activation of anti-natriuretic hormones, reduced renal blood flow and GFR, tubular cell injury, the high electrolyte concentrations due to nutrition, and replacement with hypertonic fluids. Additionally, osmoregulatory dysfunction, uremia, hypoalbuminemia, or the effects of exogenous glucocorticoids in critically ill patients may impair vasopressin. Furosemide administration may blunt renal medullary hypertonicity, leading to aquaresis [3]. During AKI, sodium excretion is reduced, leading to a net positive sodium balance [2]. Dysfunction of V2 receptors and the internalization of aquaporin 2 in intracellular vesicles reduce water reabsorption and lead to hypotonic polyuria [26]. Kidney dysfunction during AKI may also contribute to isotonic maintenance of fluids, while ongoing fluid losses may be hypotonic, and critically ill patients may lack access to water to correct this imbalance. This positive sodium balance is often observed in intensive care unit patients with AKI, where hypernatremia is associated with high total-body sodium content and normal or increased total-body water content [3].

The nature of our cohort did not allow us to determine the specific etiology of hypernatremia. However, in patients with AKI, it may be similar to that of the general population, resulting from either a net loss of electrolyte-free water or a net gain of water-free electrolytes. Notably, in our study the AKI-hyperNa group displayed clinical and biochemical signs of dehydration, such as higher urea concentrations, heart rate, body temperature, and the need for intravenous fluid adjustments, including changes to non-hyperchloremic fluids. The treatment of hypernatremia in AKI is controversial, and no randomized controlled trials have addressed this issue. There is a natural concern that administering free water to patients with ongoing AKI could exacerbate fluid overload. However, during dehydration, electrolyte-free water primarily enters the intracellular compartment, potentially reducing the risk of pulmonary edema [22].

The administration of hypotonic fluids to treat hypernatremia could be counterproductive in those with fluid overload, estimated to be close to 30% [22], which is why we emphasize the importance of ensuring the absence of fluid overload before administering volume, POCUS and VEXUS score can be an excellent strategy. Therefore, we believe that hypernatremia should be treated aggressively. Indeed, a retrospective study of 4265 hypernatremic patients found that compared to slower correction (<0.5 mEq/l/h), rapid correction (>0.5 mEq/l/h) was associated with lower mortality (31.8% versus 50.7%, *p* ≤ 0.001), shorter hospital stays, and no neurologic complications [27]. The potential benefits of rapid correction of hypernatremia would be to shorten the days with this morbidity, and thus, attenuate the risk of death, reduce neurological alterations and dehydration [13, 22–24]. The potential risks of correction would be the osmotic change and the consequences in the brain tissue, although the evidence to support this complication is scarce [27].

Our study has several limitations. The retrospective cohort design generates hypotheses and associations but does not establish causality. We did not include urinary electrolytes or osmolarity, which would have helped determine the etiology of hypernatremia. However, it is important to note that all our patients with AKI had oliguria or anuria, making urinary biochemistry interpretations unreliable. The neurological status of the patients was not captured. The specific antibiotics used were not recorded, which contained sodium and could have influenced the serum sodium of the patients. We also did not measure serum copeptin levels, which could have provided more thorough differential diagnoses. The lack of data that specify fluid management, such as detailed fluid balance, urinary output, and VExUS scores, could have affected the relationship between AKI-hyperNa and MAKE.

The number of patients included in the final analysis was relatively small. Nonetheless, the strengths of our study lie in its focus on analyzing patients with AKI-hyperNa and their association with MAKE in both the short- and long-term—objectives that, to our knowledge, have not been explored previously.

The clinical relevance of our results is that patients with AKI and hypernatremia are at an increased risk of MAKE, making them a more vulnerable group requiring a more complex approach and management. Strategies focused on correcting serum sodium should be implemented. It is possible that the precise prescription of intravenous fluids and objective evaluation of intravascular volume are key to improving patient outcomes.

In conclusion, individuals with AKI-hyperNa have a fivefold higher risk of developing MAKE in the short- and long-term compared to individuals with AKI-normalNa. The higher the serum sodium concentration and the longer the duration of hypernatremia, the greater the risk of MAKE.

## Supplementary Material

sfae419_Supplemental_File

## Data Availability

The files and data are in the physical and electronic archives of the Civil Hospital of Guadalajara Fray Antonio Alcalde and can be requested with prior authorization. All data generated or analyzed during this study are included in this article. Further inquiries can be directed to the corresponding author.
