# Supplementary material for: Acute kidney injury with hypernatremia and major adverse kidney events
Source: Clin Kidney J. 2024 Dec 18;18(2):sfae419. doi: 10.1093/ckj/sfae419 (PMC12086689; doi:10.1093/ckj/sfae419)

**Supplemental material**

**Supplemental Table 1.** Serum Sodium trajectory per day by AKI study groups.

| Day | AKI-normalNa (Na^+^ < 145 mmol/l) | AKI-hyperNa (Na^+^ > 145 mmol/l) | Total | P value |
| --- | --- | --- | --- | --- |
| 1 | 134 (128-138) | 150 (147-153) | 135 (130-142) | <0.001 |
| 2 | 134 (130-139) | 150 (143.5-152) | 137 (132-143) | <0.001 |
| 3 | 134 (131-139) | 147.5 (143-151) | 137 (133-144) | <0.001 |
| 4 | 135 (131-140) | 145 (139-151) | 137 (132-142) | <0.001 |
| 5 | 135 (131-140) | 143 (137-153) | 136 (132-142) | <0.001 |
| 6 | 135 (131-140) | 144 (139-151.5) | 136 (132-142) | <0.001 |
| 7 | 135.5 (131-140) | 142.5 (137-152) | 136 (132-142) | <0.001 |
| 8 | 135 (132-140) | 146 (139-149) | 136 (132-142) | <0.001 |
| 9 | 136 (131-141) | 143 (138.5-147.5) | 136 (132-142) | 0.0025 |
| 10 | 136 (132-142.5) | 141 (138-146) | 136 (133-143) | 0.0122 |
| Abbreviations: MAKE: major adverse kidney events. HR: Hazard ratio. LCI: lower confidence interval. UCI: upper confidence interval. | | | | |

**Supplemental Table 2**. Logistic regression model for MAKE10 by the serum sodium.

| MAKE | OR | LCI | UCI | P |
| --- | --- | --- | --- | --- |
| Serum Sodium | 1.077 | 1.040 | 1.116 | <0.001* |
| Male sex | 0.016 | 0.004 | 0.051 | <0.001* |
| Weight | 1.002 | 0.980 | 1.023 | 0.842 |
| Height | 0.192 | 0.002 | 15.34 | 0.460 |
| Systolic pressure | 1.001 | 0.985 | 1.017 | 0.849 |
| Temperature | 1.971 | 1.144 | 3.394 | 0.014 |
| Heart rate | 1.016 | 0.998 | 1.035 | 0.074 |
| Fluid adjustment | 3.177 | 0.917 | 11.00 | 0.068 |
| Antibiotic adjustment | 2.188 | 0.789 | 6.006 | 0.132 |
| Nutrition adjustment | 0.274 | 0.020 | 3.671 | 0.329 |
| Change to no hyperchloremic fluid | 1.781 | 0.277 | 11.44 | 0.543 |
| Urea | 1.005 | 1.001 | 1.009 | 0.006 |
| Abbreviations: MAKE: major adverse kidney events. OR: Odds ratio. LCI: lower confidence interval. UCI: upper confidence interval. | | | | |

**Supplemental Table 3.** Logistic regression model for MAKE10 by days spend with hypernatremia.

| MAKE | OR | LCI | UCI | P |
| --- | --- | --- | --- | --- |
| Days of hypernatremia | 1.511 | 1.222 | 1.870 | <0.001* |
| Male sex | 0.011 | 0.003 | 0.379 | <0.001* |
| Weight | 1.006 | 0.985 | 1.027 | 0.553 |
| Height | 0.592 | 0.007 | 45.90 | 0.814 |
| Systolic pressure | 1.001 | 0.984 | 1.018 | 0.889 |
| Temperature | 2.674 | 1.539 | 4.645 | <0.001* |
| Heart rate | 1.014 | 0.995 | 1.032 | 0.130 |
| Fluid adjustment | 2.544 | 0.730 | 8.866 | 0.142 |
| Antibiotic adjustment | 2.247 | 0.816 | 6.187 | 0.117 |
| Nutrition adjustment | 0.370 | 0.021 | 6.348 | 0.494 |
| Change to no hyperchloremic fluid | 0.845 | 0.120 | 5.911 | 0.866 |
| Urea | 1.004 | 1.001 | 1.008 | 0.042 |
| Abbreviations: MAKE: major adverse kidney events. OR: Odds ratio. LCI: lower confidence interval. UCI: upper confidence interval. | | | | |

**Supplemental Figure 1.** Box Plot Serum Sodium trajectory per day by AKI study groups.


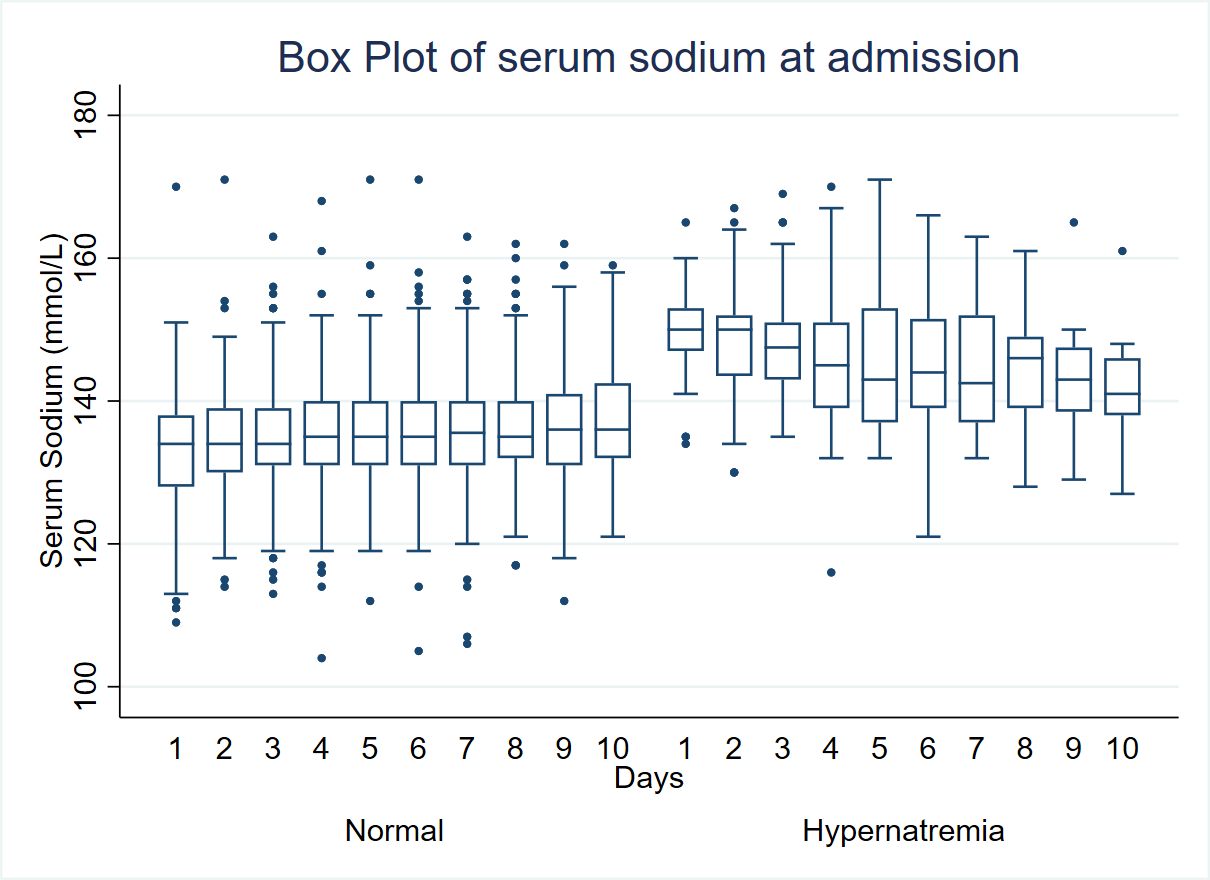


**Supplemental Figure 2.** Area under the curve for the MAKE10 by the presence of AKI-hyperNa.


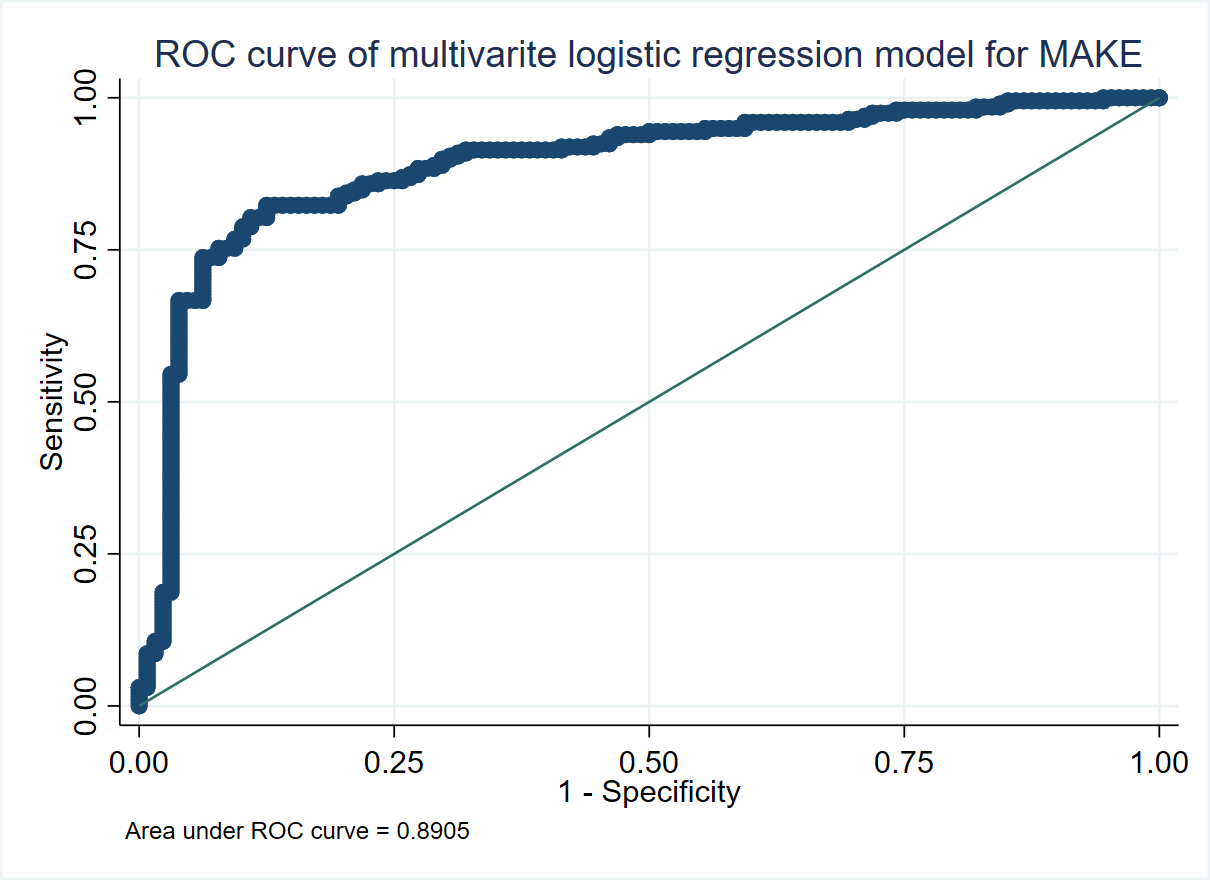


**Supplemental Figure 3**. Predictive margins of MAKE by days spend with hypernatremia.


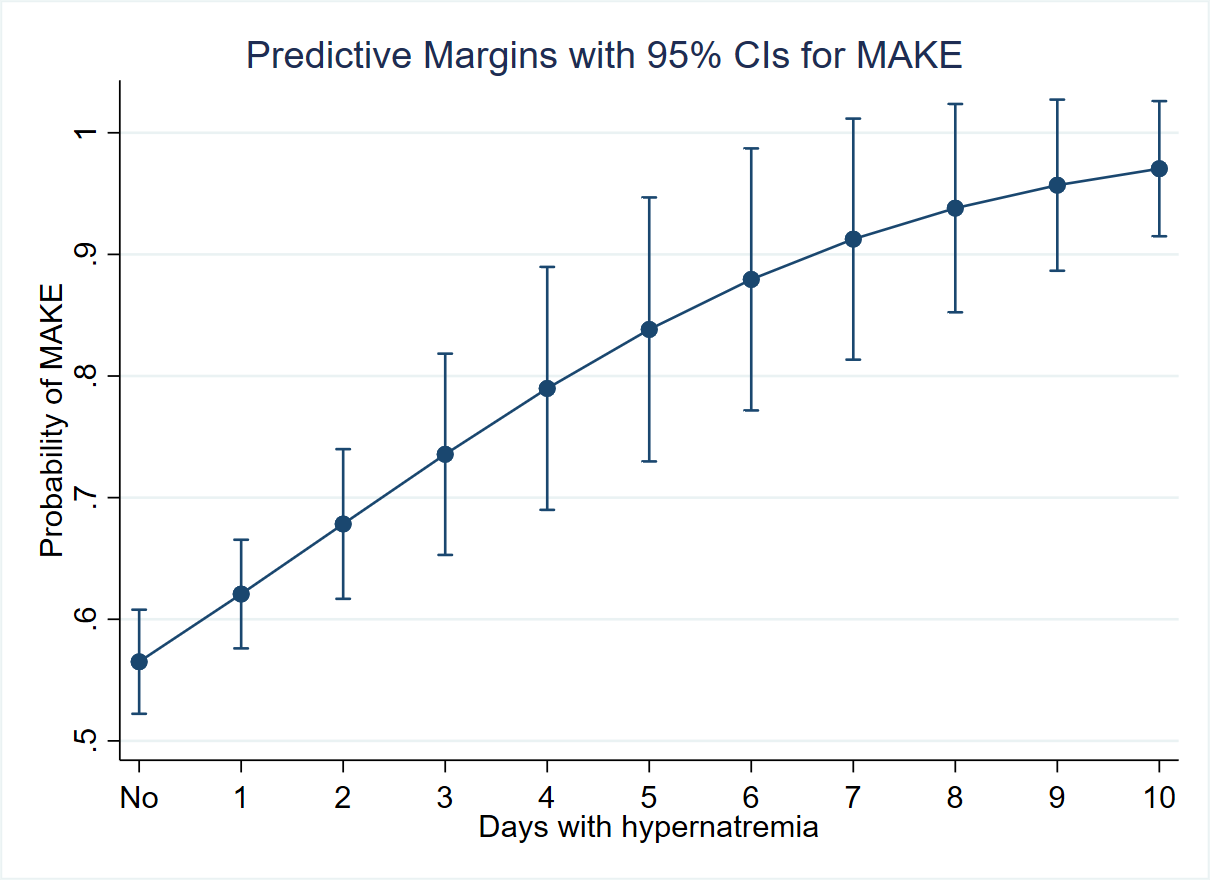

Supplement: sfae419_Supplemental_File [file sfae419_supplemental_file.docx]
